# On the Psychological Education of Medical Men

**Published:** 1848-01-01

**Authors:** 


### THE INSTRUCTION OF MEDICAL PUPILS IN MENTAL DISEASES.

Some years ago, Dr. Webster brought the question of medical pupils studying mental diseases, as a part of their ordinary education, before the profession. Additional facilities were subsequently, and in some degree in consequence of the remarks contained in Dr. Webster's pamphlet, given to pupils, as well at Bethlehem Hospital as at St. Luke's; and although the medical corporations have not hitherto thought it necessary to examine candidates coming before them as to their knowledge of the nature and treatment of insanity, nevertheless, much more attention has recently been paid in this country, than formerly, to that most important part of medical science.

In Germany, the study of insanity has also attracted considerable notice during recent years, as shown by the writings of Reil, Nostitz, Schmidt, Heinroth, and others; and even so early as 1818, Horn gave clinical instruction in Berlin on this class of disease, which he continued to do for several years afterwards. Horn was succeeded by Neumann—the latter being, in his turn, followed by Ideler, who still lectures on mental diseases in the Prussian capital. Müller also, about the same time, gave lectures on insanity at Wurtzburg. Subsequently, Autenrieth at Tubingen, Conradi at Heidelberg, Joseph Frank at Wilna, and especially Nasse at Bonn, gave clinical instruction to students on the nature and treatment of mental diseases; these lectures being illustrated by cases of insane patients received for that particular purpose into the different hospitals, of which the professors above named were the physicians. Besides the opportunities now mentioned, young medical men are admitted into the lunatic asylums of Illnau, Siegberg, and Winnenthal, to reside therein, in order to acquire practical experience in the treatment of insanity; and it is said the institutions referred to are at present well frequented.

Van der Kolk in Holland, and Guislain in Belgium, also advocate the necessity and advantage of practitioners studying mental diseases, the same as any other department of medicine. But it is in France, where the greatest progress has been really made in regard to the important question now under discussion. In that country the celebrated Pinel commenced a course of lectures, so early as 1814, on mental alienation, at his own house, which he aptly illustrated by observations on insane patients in the Salpêtrière, brought for that purpose under the notice of his pupils. Pinel was followed by Esquirol, who delivered clinical lectures on mental diseases at the Salpêtrière in 1817, which he regularly continued every year till 1826, when the government appointed him chief physician to the royal asylum at Charenton. More recently,—viz., from 1832 to 1839,—M. Ferrus lectured on insanity at Bicêtre, and also at the farm of St. Anne, an establishment subsidiary to Bicêtre, which is appropriated solely to lunatics for whom agricultural occupations are thought advisable. At both these places the lectures of M. Ferrus were generally very numerously attended. M. Leuret subsequently gave lectures also at Bicêtre in 1842; whilst M. Bottex of Lyons, and M. Rech of Montpellier, each distinguished physicians in the treatment of insane patients, have likewise contributed to the advancement of the same important object. In Paris, M. Baillarger, well known to the profession in this country, as well as in France, annually lectures on diseases of the mind, having commenced his first course in 1841, and he frequently illustrates the subject under discussion by cases selected from the insane patients resident in the Salpêtrière. M. Falret, another distinguished physician, and attached to the Salpêtrière, also gives a public course of lectures on insanity, which he has continued annually since 1843, with much success.

Although considerable progress has undoubtedly been recently made in England in this department of medical education, for many years a retrograde movement had actually taken place, particularly subsequent to the period when the celebrated and learned physician, Dr. William Beattie, was attached to St. Luke's Hospital, who states in the preface to his "Treatise on Madness," published in 1758, that "by an unanimous vote, the governors (of St. Luke's) signified their inclination of admitting young physicians, well recommended, to visit the hospital, and freely to observe the treatment of the patients confined." In order that the attendance of the pupils might be rendered more instructive, Dr. Beattie also "offered to the perusal of the gentlemen who honoured him with their attendance the reason of those prescriptions which were submitted to their observation." Of late, however, the tide has happily turned in the right direction, especially since so many new lunatic asylums have been, and will be soon, erected throughout the country, which must require a larger number than heretofore of medical practitioners actually conversant with mental alienation. Besides Sir A. Morison, who, for many years, was the only lecturer on diseases of the mind in London, Dr. Conolly has given repeated courses of lectures on insanity at Hanwell since 1842. Dr. A. Sutherland has likewise lectured and given clinical instruction at St. Luke's, when the doors of that institution were re-opened in 1842 for the admission of pupils, after having been closed during a great number of years, notwithstanding one of the objects proposed at the foundation of that charity, according to the original address in 1751, was "of introducing more gentlemen of the faculty to the study and practice of one of the most important branches of physic." Again, during last summer, Dr. Hitchman has delivered a few lectures on the pathology of mental disease at Hanwell. Besides the means above stated of obtaining psychological instruction, several of the provincial lunatic asylums receive a limited number of young medical men as resident pupils, for the purpose of obtaining information and practical experience respecting the nature and treatment of mental alienation. However, until the medical colleges of the empire absolutely require from all those applying for their diploma, proof that they possess adequate knowledge to entitle them to undertake the management of patients afflicted with insanity, quite as much as to treat diseases affecting the physical frame, in all probability the progress made towards the attainment of the object now alluded to will be slow, although, to use the words contained in the pamphlet published by Dr. Webster, in 1842, on this really important question—"Few diseases impose so much responsibility upon the attending physician as mania, whether the case be considered in a medical or legal sense, and the worst consequences may sometimes result to the patient, should even a trifling mistake be committed by the attendant. Examples of errors in judgment, of a very painful description, might be quoted, from books and public records, to show the important consequences sometimes resulting to a fellow-creature, from inattention to the premonitory symptoms of lunacy. But it is unnecessary to enter into details, as it will be readily allowed by those conversant with the subject, that scarcely any complaint to which mankind is liable requires more to be studied than a disease of the mind, so as to alleviate, when unable to cure, the attacks of such a deplorable affliction to humanity, which destroys, as it were, the moral existence of a fellow-creature, although physical life, with all its wonderful functions, still continues to animate this mortal frame."

---

### Medical Intelligence.

